# Improving career guidance accuracy through EEG-based vocational interest assessment and self-reported RIASEC profiles

**DOI:** 10.3389/fnins.2026.1883915

**Published:** 2026-08-21

**Authors:** Mihai Cristian Orzan, Elena Goga, Dănuț Trifu, Mihai Diaconescu, Mihaela Constantinescu

**Affiliations:** 1 School of Marketing, Bucharest University of Economic Sciences, Bucharest, Romania; 2 Laboratory of Neuromarketing Consultapro, Bucharest, Romania

**Keywords:** career guidance, educational services, electroencephalography, John L. Holland, neuroimaging technique, neuroscience, occupational interests, RIASEC framework

## Abstract

This study investigates the potential of electroencephalography-based neurometrics to enhance vocational interest assessment within John L. Holland’s RIASEC framework, which classifies occupational preferences across six dimensions: Realistic, Investigative, Artistic, Social, Enterprising, and Conventional. The research compares occupational interest profiles obtained through a widely used self-report instrument with profiles derived from EEG-based responses recorded while participants engaged with the same assessment items. By integrating neurophysiological measures into vocational profiling, the study explores whether neurometric indicators can provide additional insight into underlying occupational preferences beyond consciously reported interests. The O*NET Mini Interest Profiler was administered to 33 participants while EEG data were simultaneously recorded, generating 990 paired observations for comparison between self-reported and neurometric-derived scores. The findings reveal a strong overall association between the two assessment approaches, suggesting substantial convergence between declared interests and EEG-based indicators. However, the analysis also identifies meaningful discrepancies in individual RIASEC profiles. These differences frequently led to distinct occupational recommendations, underscoring the practical significance of even small variations in vocational interest assessment, particularly given the extensive mapping of RIASEC profiles to more than 900 occupations in the O*NET system. The results suggest that EEG-based neurometrics may complement traditional self-report measures by capturing less explicit or less consciously articulated dimensions of vocational preference. In a context marked by rapid labour market transformation and increasingly diverse educational pathways, more refined methods for identifying individual occupational interests may improve career guidance and better align personal preferences, educational choices, and professional trajectories. The study also discusses implications for educational institutions seeking to optimise program offerings in support of sustainable education and proposes directions for future research on integrating neurophysiological methods into vocational assessment.

## 1 Introduction

High-potential students invest time and financial resources in educational pathways aligned with their career goals, supported by career counsellors, institutions and extensive relevant data. Occupational interest inventories are widely used and becoming increasingly important, especially for top candidates, compared to skills or aptitude assessments (Nye et al., 2012). This trend is strongly supported by labour market disruptions and the emergence of entirely new professions, making interest-based evaluations particularly valuable for navigating novel career opportunities. Moreover, these occupational interests would be a constant factor in all the educational choices the professionals would make throughout their lives, well beyond formal graduate or postgraduate studies.

This is the point at which Holland’s model proves its utility by providing valuable insights into person-environment fit, thereby sustaining future-informed educational and career decisions. Over the past three decades, Holland’s theory has evolved from a simple description of six personality types and the corresponding environments into a more comprehensive, evidence-based framework. Nowadays, it focuses on three key elements: individuals, the environments they are part of, and the ways these two interact (Holland, 1997; Rocconi et al., 2020). In the field of higher education, Holland’s framework focuses on students, the subjects they choose to study, and how well these choices match their interests and characteristics (Smart et al., 2000; Smart et al., 2009).

The key idea behind Holland’s theory is that individuals can be classified using one or more of six personality types: Realistic, Investigative, Artistic, Social, Enterprising, and Conventional. The second part of Holland’s theory focuses on the environment. The six types of environments correspond to the six personality types, and each tends to support and encourage the characteristics that fit its matching personality profile (Holland, 1997; McDaniel and Snell, 1999). In higher education, academic disciplines can be seen as types of environments. The third component of Holland’s theory focuses on how personality types and environments interact or how well they fit together (Šverko et al., 2006; Pike, 2006).

Fritzsche et al. (2002) demonstrate in their paper that the Holland framework plays an important role in proving how personality relates to performance. The results suggest that personality does not predict performance in the same way across all situations, but rather depends on how well a person’s traits fit the demands of their work environment. This points out the value of considering vocational context when using personality measures, both for improving theory and for practical applications such as employee selection and career guidance.

Holland’s RIASEC framework (Realistic, Investigative, Artistic, Social, Enterprising, and Conventional) (Holland, 1959, 1997) is adopted by many occupational interest inventory providers. Today, the most relevant is the significant development by O*NET (Occupational Information Network) for the U.S. Department of Labour (Rounds et al., 2021). The Occupational Information Network is the most comprehensive database of worker attributes and job characteristics. Spanning 900 occupation profiles and over 55,000 jobs, each profile is a reliable source of current information. Portions of every profile are refreshed annually, while details such as job titles and technology skills are updated even more frequently. As new tools and methods emerge, O*NET continues to enhance and expand its data collection.

Beyond the conceptual and operational developments, the main merit of this endeavour, from the researchers’ point of view, lies in the attempt to practically link the RIASEC profiling to over 900 occupation types in a timely and documented manner. It is this huge effort and data volume generated that may be leveraged to improve individual profile assessment, leading, as shall be argued, to improved results for learners seeking educational services and for the institutions that may provide them.

The educational services market is also highly dynamic and will most likely continue this trend in the foreseeable future. Beyond the standard courses within established university sites, a learner may choose online classes from the same institutions, grouped in a certified program or not; a great number of independent online providers of very narrow and specific skillsets are widely available, while other organisations (e.g., Chartered Financial Analyst Institute) compete with the traditional ones for setting the industry standards.

The Holland test is based on self-reported responses, asking participants to imagine themselves performing various activities and to indicate how appealing they find each one. The basic assumption is that individuals can provide meaningful information about their own interests, preferences, and perceived strengths. Based on these responses, a profile is generated across the six RIASEC dimensions: Realistic, Investigative, Artistic, Social, Enterprising, and Conventional, providing a general picture of the participant’s vocational interests (Holland, 1997).

The self-response process is typically structured into several stages. In the first step, participants are asked to list occupations they have considered, admired, or imagined for themselves. These responses provide valuable insights into their career interests and aspirations. According to Holland (1997), such occupational preferences or “career daydreams” can be particularly informative, as they often reflect underlying vocational interests even before the formal assessment is completed.

In the second step, individuals indicate whether they would enjoy participating in various activities. These activities are grouped according to the six RIASEC types. In the following stage, participants are asked to evaluate their own abilities and achievements. This part of the assessment is based on the idea that interests and perceived competencies are closely related, as individuals often develop stronger skills in activities they enjoy and engage in regularly. In the fourth stage, participants are presented with a list of occupations and asked to indicate to which extent each career interests them. Their responses provide additional insight into their vocational preferences and help refine the identification of their dominant RIASEC dimensions (Holland, 1997; Reardon and Lenz, 1999). The fifth stage focuses on participants’ self-perceived abilities. Respondents are asked to evaluate their skills in areas related to each RIASEC dimension, typically by comparing themselves to others of a similar age. These self-ratings complement the interest-based measures and provide a more comprehensive picture of the individual’s vocational profile. In the sixth phase, all responses are compiled and scored. Points are summed for each RIASEC category, after which the six categories are ranked from highest to lowest. The three highest scored categories are then combined to form the individual’s Holland Code (Gottfredson and Holland, 1996; Holland, 1997, 2016).

This code reflects the individual’s main pattern of vocational interests and is typically used to identify educational paths and occupations that align with a similar code. As a self-report inventory, it offers several advantages, including ease of administration, low cost, quick scoring, and direct access to individuals’ interests and preferences. At the same time, it also has certain limitations. Responses may be influenced by self-perception, and individuals may sometimes answer in line with perceived expectations rather than real preferences. In addition, limited exposure to specific activities or careers can shape responses, and self-ratings do not always accurately reflect actual abilities.

To address the limitations associated with self-reported data, this study incorporated in its research EEG neuroimaging techniques. Previous findings (Vecchiato et al., 2011; Nielsen, 2016; Byrne et al., 2022) suggest that metrics derived from raw EEG signals can provide a more precise indication of the level of attractiveness or engagement generated by different activities compared to self-report measures.

### 1.1 Neuromarketing—neuroscience applied to consumption

Here comes the turning point at which neuromarketing techniques can enhance the results achieved when the RIASEC framework is applied to the subjects of research.

Initially introduced by Ales Smidts (2002), the term “neuromarketing” describes an applied field situated within the broader domain of neuroeconomics, which itself integrates insights from psychology, economics and neuroscience. Consumer neuroscience combines tools from neuroscience and consumer psychology to develop a more comprehensive neuropsychological approach to understanding consumer behaviour (Plassmann et al., 2012). Thus, neuromarketing incorporates various neurological and physiological techniques that enable deeper insights than those typically obtained through conventional marketing research approaches (Meyerding and Mehlhose, 2020; Oberg, 2023). The application of neuroscience methods in marketing research is commonly referred to as neuromarketing, also known as consumer neuroscience (Genco et al., 2013). It focuses on using insights from brain science to understand better how consumers respond to marketing stimuli (Lin et al., 2018).

Optimism regarding the potential of neuromarketing has been linked to the expectation that it can provide marketers with consumer insights difficult to obtain through traditional approaches (Bakardjieva and Kimmel, 2017). Traditional marketing methods, such as surveys and focus groups, rely on self-reports and introspection to understand consumer preferences and have been widely used for this purpose. However, neuroimaging can identify these preferences even before consumers become consciously aware of them (Ariely and Berns, 2010). Using neuromarketing tools, implicit reactions can be examined and deeper insights gained into consumers’ experiences, branding materials, and product design that resonate with consumers at a subconscious level (Fugate, 2007; Agarwal and Dutta, 2015; Singh et al., 2024).

Thus, neuromarketing provides a useful framework for evaluating commercial effectiveness through indicators such as attention, novelty, emotional engagement, memory retention and purchase intention. Its increasing use in consumer research reflects a growing recognition of the importance of both emotional and cognitive processes in shaping decision-making. In their study, Traymbak et al. (2024) highlight the central role of emotion in guiding purchasing decisions, particularly among Indian consumers, where affective states strongly influence behaviour. Overall, higher levels of emotional arousal appear to enhance engagement, suggesting that more intense consumer experiences are associated with stronger behavioural responses.

Neuromarketing research investigates consumers’ subconscious responses to different marketing stimuli (Bajaj et al., 2023; Bhardwaj et al., 2023). According to Singh et al. (2024), deeper insights from neuromarketers help companies design experiences, products, marketing campaigns, and branding that connect more naturally with consumers on a subconscious level. Neuromarketing, also known as neuroscience applied to consumption (Ramsøy, 2015; Pagan et al., 2024; Traymbak et al., 2024), makes it possible to obtain deeper and more objective information about consumer behaviour than traditional marketing methods do (Singh and Kumar, 2018; Pagan et al., 2024; Bucea-Manea-Țoniș et al., 2024).

In their study, Puprediwar and Tapas (2024) highlight the growing role of neuromarketing as a complement to traditional consumer research methods, offering deeper insights into subconscious processes that shape decision-making. While its adoption remains far behind classical marketing research methods, the findings suggest increasing recognition of its potential to enhance the accuracy and richness of consumer insights. At the same time, several challenges persist, including methodological limitations, ethical concerns and issues related to accessibility and cost. These constraints indicate that neuromarketing should not replace conventional approaches but rather be integrated with them to provide a more comprehensive understanding of consumer behaviour (Puprediwar and Tapas, 2024). As the field continues to develop, combining neuromarketing with traditional research methods seems essential for gaining a more reliable understanding of consumer behaviour (Bhardwaj et al., 2024).

Sieber (2019) highlights important ethical challenges arising from the growth of neuromarketing. While advances in neuroscience offer new ways to understand better and influence consumer behaviour, they also raise questions about where the line lies between persuasion and manipulation, especially at a subconscious level. Current legal and ethical frameworks are not fully equipped to address these issues, particularly in protecting individuals’ cognitive and emotional autonomy. This points to the need for clearer guidelines, greater transparency and stronger safeguards.

Referring to the area of interest for this study, many of educational providers already use neuromarketing approaches, from getting insights on the decision criteria strength to promotional messages optimisation (Kenning and Linzmajer, 2011; Smidts et al., 2014), as there is no debate on EEG measures to predict consumer decisions and to assess cognitive and emotional states way better than the traditional, self-reported methods (Ramsøy et al., 2018; Byrne et al., 2022). At the core of this paper is an essential neuromarketing approach for educational service providers: how can “consumers’” preferences be better assessed? Neuromarketing, through its methodological tools, contributes to greater alignment between educational offerings and learners’ needs and preferences.

### 1.2 EEG: reliable neuroimaging technique used in marketing research

Electroencephalography (EEG) is a non-invasive neuromarketing technique that records electrical activity at the brain’s surface via scalp electrodes (Pagan et al., 2024). The electrodes are strategically positioned on the scalp to capture neural activity from different brain areas (Mahamad et al., 2019; Aldayel et al., 2020). There is increasing interest among researchers and marketers in the use of EEG due to its high temporal resolution, relatively low cost and ease of use (Ariely and Berns, 2010; Kolev, 2019). As Pagan et al. (2024) demonstrated, EEG is widely used in studies of preferences (Khushaba et al., 2013), advertising, the country-of-origin effect, price, brand, and decision-making (Wang et al., 2014). Hakim and Levy (2019) suggest that EEG is a valuable tool with considerable future potential, especially when it is combined with complementary methods.

The key limitation noted in the historical development of the RIASEC framework is the establishment of the vocational interest profile based solely on the user’s self-report. Still, neuromarketing studies have repeatedly shown the limited ability of this type of approach to accurately capture the respondent’s real preferences (Venkatraman et al., 2015; Cherubino et al., 2019).

Replacing the self-reported responses with neurometric scores derived from EEG data has been proven to be more than three times more effective, offering improved accuracy and reliability in measurement (Byrne et al., 2022; Mashrur et al., 2022). In their systematic review of EEG measures and machine learning in neuromarketing, Byrne et al. (2022) indicate that combining these technologies yields highly accurate predictions of consumer preferences and purchasing behaviour, often overcoming the limitations of self-report methods. The integration of machine learning with EEG data significantly improves predictive accuracy for customer preferences, with accuracies ranging from 60% to over 90%. Nielsen (2016) provides valuable information in his experiment. He shows that the predictive value of questionnaires is around 20%, whereas that of EEG is around 60%. The best results were obtained when using the combination of EEG and questionnaires, with a predictive value of almost 80%. In 2022, Mashur et al. reported EEG data accuracy of 84–87% in their study, and Byrne et al. reported accuracy of 85–94% for the EEG metrics developed with the Emotiv EPOC X EEG headset. The research team of this study used an Emotiv EPOC X EEG headset to collect EEG metrics, as well.

While the acquisition of raw EEG signals follows standardized recording procedures, the processing and interpretation of these data can vary depending on the analytical methods and software employed. In the present study, the Interest (Val) metric provided by Emotiv was used as the primary measure of participant engagement with the presented stimuli.

Briefly, metrics of this type are developed by feeding various artificial neural networks with hundreds or thousands of data sets formed by the raw EEG recordings (changes in post-synaptic electrical potentials, measured in microvolts by each EEG sensor) and the emotional or cognitive state as assessed in controlled conditions via publicly available methodologies (Pacheco et al., 2018; Alkhomsan et al., 2024). If a neural network produces stable correlations between the sensors’ raw values and the assessed state values in the test stage (other large data-sets) we can use it in future projects. Currently, these correlations are of more than 90% accuracy (Byrne et al., 2022). This approach goes far beyond the traditional EEG indicators commonly used to predict preferences, such as the Frontal Asymmetry Index (regardless of the frequency band considered) (Ramsøy et al., 2018), the Late Positive Potential (LPP) or Theta Band Power. Metrics that integrate data from multiple brain regions, can attenuate errors arising from various neuronal populations being activated in other circuits than the ones of interest. At the same time, they are less affected by the information loss introduced by the Fourier transformation (Kiebel et al., 2008; Abreu et al., 2018).

As demonstrated in their paper, Mizrahi et al. (2025) show that EEG data can predict psychological styles traditionally defined by questionnaires. This implies that EEG can replace or improve self-reported methods, especially for psychological states (Li et al., 2023).

Van der Linde et al. (2022) demonstrate the value of EEG as an effective tool for examining implicit psychological processes. The findings show that EEG-based measures can capture neural responses that are not always accessible through self-report methods; they highlight the differences between conscious reporting and underlying automatic reactions. By providing objective data on brain activity, EEG complements traditional questionnaires and helps in reducing biases associated with self-reporting. Overall, the results support the efficiency of EEG in revealing implicit cognitive and emotional processes with a level of precision difficult to achieve with behavioural measures alone (Constantinescu et al., 2019).

Yim et al. (2023) suggest in their paper that EEG can provide a clearer, more objective picture of mental workload than people’s self-reports, which often fail to capture cognitive effort fully. By revealing neural activity that may not be perceived in a conscious state, EEG offers a more reliable way to assess mental demands in data visualization tasks. The results of their study support the use of EEG as a useful and reliable neuromarketing tool.

In their systematic review, Esteves and Vourvopoulos (2026) highlight the limitations of self-report methods and note that only a minority of studies include objective measures, even though these are essential. The conclusions of their study support the need for the EEG technique as a complementary objective measure in future research.

### 1.3 Research questions

This research builds on previous attempts to use neuroimaging tools to improve career assessment (Rasheed et al., 2019). To date, researchers have demonstrated that neuroimaging techniques provide additional useful information beyond self-reported data (Gurres et al., 2021).

Motivated by evidence that EEG can act as an alternative predictor for career interests and can be relevant because congruence impacts future career outcomes (Batista and Gondim, 2023), the research team sets off from the point where Gurres et al. (2021) using magnetic resonance tomography and the possibilities of Voxel-Based Morphometry in their research, suggest for further research the validation of Holland’s tools by using other neuroimaging techniques, as objective research methods.

In support of the above, the work of Schröpel et al. (2025) on the relationship between vocational interests and career decisions is cited. As a direction for further research, they emphasise the need to explore the mechanisms underlying these relationships in greater depth and to use objective methods.

In conclusion, previous research has suggested that objective neuroimaging techniques may complement traditional self-report measures in vocational assessment. Starting from these findings and the identified research gaps, this study addresses the following research questions:

*RQ1*: To what extent do EEG-derived vocational interest scores correlate with self-reported scores obtained through the O*NET Mini Interest Profiler?

*RQ2*: Do EEG-derived metrics generate RIASEC vocational profiles that differ from those obtained through traditional self-report scores?

*RQ3*: Can EEG-based neurometric measures provide additional information that may improve the accuracy of vocational interest evaluations and career guidance compared to self-report methods alone?

By exploring these questions, this study seeks to determine the potential contribution of EEG-based neurometrics to vocational assessment and what role they may play in supporting educational and career decision-making.

### 1.4 Research objective and contribution

Despite the growing adoption of Holland’s RIASEC framework in vocational guidance and the increasing evidence that is supporting the predictive value of EEG-based neurometrics, only a limited number of studies have examined whether neurophysiological measures can enhance the assessment of vocational interests and improve career recommendations. Previous research has emphasized the importance of validating Holland-based assessment tools using objective neuroimaging techniques in addition to conventional self-report measures.

Therefore, the main objective of this study is to investigate whether vocational interest profiles based on EEG metrics differ from those obtained through a conventional self-report RIASEC inventory. To be more precise, this study compares self-reported and EEG-based occupational interest assessments in order to determine:

1. The degree of convergence between the two approaches;
2. Whether EEG-derived profiles lead to different Holland codes and occupational recommendations;
3. The potential value of integrating EEG-based neurometrics into career guidance and educational decision-making.

By addressing these objectives, this research contributes to the emerging literature on neuroscience-based vocational assessment and provides empirical evidence regarding the complementary role of EEG measures in improving the accuracy of career guidance.

## 2 Materials and methods

### 2.1 Research design

This paper presents a cross-sectional study. After recruiting participants in 2 important academic cities in Romania (Bucharest and Iasi), EEG neuroimaging and the Mini-IP Interest Profiler questionnaire were administered to each subject in the study. Participants completed self-reported occupational interest assessments, and EEG data were recorded at the same time.

### 2.2 Participants

A total of 33 respondents were selected for this research, including 16 females and 17 males. Their ages ranged from 21 to 58 years. There were 5 females between 20 and 29 years, 7 females between 30 and 39 years and 4 females between 40 and 59 years. Regarding the males, there were 6 subjects in the interval from 20 years to 29 years, 4 subjects in the interval from 30 years to 39 years and 7 subjects from 40 years to 59 years.

They answered the questions while wearing the Emotiv Epoc X EEG headset. All the subjects that took part in this study signed, at the beginning, an informed consent for the manipulation of the data collected during this research.

### 2.3 Data acquisition

EEG data were collected using the Emotiv EPOC X system. The device was equipped with 16 sensors positioned according to the international 10–20 System, with electrodes placed at AF3, AF4, F3, F4, F7, F8, FC5, FC6, T7, T8, P7, P8, O1, and O2. In addition, the headset included a reference electrode located at M1 and a noise-cancellation electrode located at M2. The system was used to record raw EEG signals throughout the experiment. An EmotivPRO license enabled the collection of the raw data and provided access to the metric of interest used in the analyses performed in this study.

Researchers adhered to O*NET’s original instructions, asking respondents to imagine themselves performing each activity they were evaluating. Participants were given 6 s to read each activity and 10 s to both imagine and rate it, allowing the researchers to collect self-reported ratings alongside the corresponding EEG-derived metrics.

The resulting datasets were then used to:

- Assess the overall correlation across the 990 paired observations;
- Evaluate correlations at the individual respondent level;
- Compute standard Holland codes for each participant based on both self-reports and EEG metrics;
- Examine the degree of congruence between the two sets of codes, as well as the implications of discrepancies.

The researchers used the Mini Interest Profiler (Mini-IP) developed by O*NET (Rounds et al., 2016; Lewis, Morris, and National Centre for O*NET Development, 2016), which has been used by the U.S. Department of Labour in various forms since 1999 and by specialists in career guidance.

The Mini-IP Interest Profiler consists of 30 questions, five of which count for the final score of each of the RIASEC (Realistic, Investigative, Artistic, Social, Enterprising and Conventional) occupational interest categories as originally proposed by Holland (1959) and refined over the years by numerous authors (Holland, 1997; Lewis and Rivkin, 1999). Although versions of 180 questions (Interest Profiler Long Form) and 60 questions (Interest Profiler Short Form) were released by the same organization, researchers used the 30-question Mini-IP, encouraged by its validity evidence from Rounds et al. (2016).

No attempt was made to calibrate this inventory to the Romanian audience (Su et al., 2014; Rounds et al., 2016). Still, the researchers changed the original 1–5 scale into a 1–10 scale for four reasons:

- The 1–10 scale is more commonly used in Romania.
- In contrast to the 1–5 scale, theoretically, it does not allow for perfectly neutral answers.
- It enables finer differentiation between various levels of professional interest.
- The “Interest” metric developed by Emotiv provides scores on a 1 to 100 scale, much easier to compare with a 1 to 10’s results than with a 1 to 5’s.

The original O*NET Mini Interest Profiler uses a five-point Likert scale ranging from 1 (“Strongly Dislike”) to 5 (“Strongly Like”). In the present study, this response scale was linearly rescaled to a 1–10 format to facilitate direct numerical comparison with the EEG-derived “Interest (Val)” metric, which is also reported on a 1–10 scale, while maintaining the ease of use for respondents as intended by the O*NET developers. Linear transformations of Likert-type response scales preserve the relative measurement properties of the data because they do not alter the ordering of responses or the relationships among variables, provided that the questionnaire content and the underlying construct remain unchanged (Boone and Boone, 2012; Boone, 2025). Accordingly, the present adaptation did not modify the questionnaire items, their wording, the underlying RIASEC constructs or the scoring procedure used to derive vocational profiles. Consequently, the relative ordering of participants’ responses and the interpretation of the six vocational interest dimensions were preserved, ensuring direct compatibility between self-reported and EEG-derived interest scores. Because the analyses focused on within-sample comparisons between self-reported and EEG-derived scores rather than on comparisons with published O*NET normative data, the scale transformation is not expected to affect the psychometric interpretation of the results within the context of the present study.

The difference between 4 and 3 on the original scale is not the same as the one between 8 and 6 on the modified one, especially as the 3 is a perfectly neutral answer while 6 should design a slightly positive one. As a result, the findings of this study do not directly rely on the documented effectiveness of O*NET. However, the main aim was to investigate the differences between vocational profiles and careers advocated by self-answers and EEG metric scores. In this context, adopting a 1–10 scale, much more familiar to respondents, answered the O*NET prescription for less cognition filtered feedback.

However, as the adapted 1–10 response format was not subjected to a separate psychometric validation, comparisons with published O*NET normative data should be interpreted with caution. The main purpose of this research was to examine the degree to which self-responses differ from EEG-assessed attractiveness, leading to different RIASEC profiles and proposed professional pathways. Future studies should formally evaluate the measurement equivalence of the rescaled instrument using larger validation samples.

### 2.4 Neuroscience methods contribution

An important methodological consideration concerns the use of the Emotiv “Interest (Val)” metric as the primary EEG-derived measure. Although this metric is generated using artificial neural network models trained on large EEG datasets, the underlying algorithms and feature extraction procedures are proprietary and therefore not publicly available for independent examination. Nevertheless, the present study did not seek to validate the proprietary algorithm itself but rather to evaluate whether the resulting EEG-derived interest scores exhibit meaningful relationships with established self-report measures of vocational interests. The significant correlations observed between the EEG-derived and self-reported RIASEC profiles provide preliminary evidence supporting the practical utility of the metric within the context of vocational assessment.

Proprietary metrics have historically been labelled as black boxes, prominent authors rightfully complaining of their lack of transparency (Ramsøy, 2015). While the current level of transparency is still limited, there have been encouraging advances in recent years:

1. Even though developers of proprietary metrics do not fully disclose the specifics of the techniques used to elicit and assess the emotional and cognitive states for generating the data needed for training and testing the AI network, its characteristics (layers, numbers of neurons, architecture), or results in the testing stage, they do acknowledge the overall method and data-sets sizes.
2. Several meta-studies documented the effectiveness of such metrics (Byrne et al., 2022; Fernandes et al., 2024).
3. Some authors (Trifu et al., 2024), without revealing more than the rightful owners allow, performed a reverse engineering on several metrics, relating the metrics scores with the raw EEG readings via their own AI networks with more than 80% accuracy in the test stage (readings from AF3, AF4, F3, and F4 had significant weights for the Interest metric).
4. Finally, efforts are made to develop such metrics within academic framework, allowing for increased transparency (Keil et al., 2014; Pernet et al., 2011).

Oversimplifying, rather than disregarding all the other readings considered relevant by the AI model after being trained and tested on large data sets, and processing only the frontal and prefrontal data, a more fruitful avenue may be to revert to the areas that appear relevant and examine them in separate analyses.

Future research should further validate these findings by combining proprietary EEG-derived metrics with analyses based on established, open neurophysiological indicators. In particular, raw EEG recordings could be analyzed using conventional measures such as Frontal Alpha Asymmetry (FAA), which has been extensively associated with motivational tendencies, approach–avoidance behaviour and affective preferences (Harmon-Jones and Gable, 2018; Sabu et al., 2022), as well as other well-established EEG markers including frontal theta activity (Messel et al., 2021), event-related potentials (e.g., Late Positive Potential) (de Tommaso et al., 2020). Comparing these transparent neurophysiological indicators with the outputs generated by proprietary algorithms would provide stronger evidence regarding the validity, reliability, and interpretability of EEG-based vocational interest assessment.

## 3 Results

Across 990 data pairs from 30 occupational interests measured via self-report and EEG metrics for 33 participants, the analysis yielded a correlation coefficient of 0.6381, which is statistically significant at all conventional confidence levels (Cohen, 1988; Luck, 2014; Field, 2018).

In Table 1, the correlation coefficients for each person between his or her self-reported scores and the metric’s values are presented, followed by the computed t-values for testing the significance of the correlation coefficients. The codes are the ones commonly used within the RIASEC framework, listing the first three categories as total points obtained (first letter stands for the category with the highest total points) (Holland, 1997; Nauta, 2010). A fourth or fifth letter was added when two or three categories had identical third top score.

**Table 1:** Respondents and main statistics.

No.	Sex	Age	Correlation (rounded)	t−computed^(1)^	Self-response code^(2)^	EEG metric code^(3)^	Codes match^(4)^
1	f	21	0.75	6.0000	A_SE	E_SA	Weak
2	m	21	0.62	4.1814	I_ER	R_SI	Weak
3	f	22	0.57	3.7143	I_AES	C_EI	Weak
4	f	23	0.57	3.6942	A_SI	A_SI	Perfect
5	m	23	0.47	2.8176	E_CR	R_SCE	Weak
6	m	24	0.68	4.9075	R_AE	E_RC	Weak
7	f	26	0.79	6.8182	I_SA	I_AS	Strong
8	m	27	0.60	3.9686	C_IR	C_RS	Strong
9	m	28	0.52	3.2214	E_CR	R_EC	Weak
10	f	29	0.66	4.6487	E_IR	E_SI	Strong
11	m	29	0.77	6.3859	R_ES	C_ER	Weak
12	f	31	0.64	4.4074	A_SI	E_SA	Weak
13	m	31	0.76	6.1877	E_RC	E_RC	Perfect
14	f	32	0.63	4.2926	S_EC	C_ES	Weak
15	f	33	0.65	4.5260	S_CE	C_AS	Weak
16	m	35	0.44	2.5927	E_IC	E_RS	Strong
17	f	36	0.60	3.9686	S_IA	I_CS	Weak
18	f	37	0.71	5.3351	I_AS	E_SI	Weak
19	m	37	0.68	4.9075	R_CE	I_ER	Weak
20	m	38	0.69	5.0443	C_ER	C_ER	Perfect
21	f	38	0.70	5.1867	S_AI	E_SA	Weak
22	f	38	0.74	5.8217	S_EC	E_SC	Weak
23	f	40	0.75	6.0000	E_AS	E_AS	Perfect
24	f	40	0.51	3.1373	R_SACE	S_ER	Weak
25	m	41	0.51	3.1373	R_EC	R_ES	Strong
26	m	42	0.71	5.3351	C_ES	S_RC	Weak
27	m	44	0.63	4.2926	C_RS	C_RS	Perfect
28	m	44	0.65	4.5260	R_SI	R_IC	Strong
29	f	45	0.59	3.8667	S_CAE	S_AC	Strong
30	m	47	0.77	6.3859	R_CS	R_CE	Strong
31	m	52	0.75	6.0000	R_SCE	R_SE	Perfect
32	f	56	0.73	5.6519	C_RE	C_RS	Strong
33	m	58	0.46	2.7413	R_AC	R_AI	Strong

As shown in Table 1, individual correlation coefficients ranged from 0.44 to 0.79, all significant at the 0.05 level. Furthermore, for 28 out of 33 participants, significance was also achieved at the 0.001 level.

According to Holland’s (1997) codes, the two assessment methods produced identical results for six participants, and an additional 10 participants shared the same dominant interest category. Notably, in no case did the dominant category derived from self-reports fall outside the top three categories identified by EEG-based metrics.

No statistically significant relationship was found between age and the correlation coefficients (Salthouse, 2010; Grady, 2012). The correlation coefficient between respondents’ age and metric—self-reported scores stands at 0.047, statistically insignificant. Despite the much larger data sets used, 380 pairs of metric and self-report scores for the 16 female participants and 410 for the 17 male participants, no statistically significant difference was observed between the two groups, with the z_-difference_ standing at 1.920335 (*p*-value of 0.0548).

In Table 1 the column for t−computed^(1)^ critical t values for a two-tailed test are *t*_(0.05, 28)_ = 2.0484 and *t*_(0.001,28)_ = 3.6739. In the columns *Self-Response Code*^(2)^ and *EEG Metric Code*^(3)^, Holland’s codes usually come as the first three scores of the RIASEC interest categories. When equal scores were obtained for the third-place category, they were all included in alphabetical order. As for the column *Codes Match*^(4)^, the codes’ match is considered “Perfect” when the first three categories are the same in the same order, regardless of the last three. It is “Strong” when at least the first, “dominant” category is the same, and “Weak” when the dominant category, as assessed by the two methods (self-response scores and EEG metric scores), differs.

A contrasting pattern emerged when comparing metric–self-response correlations across the RIASEC model categories and the 30 rated items. The results are presented in Tables 2, 3. Metric–self-response correlations are statistically significant across all six categories, though their magnitudes vary considerably. At the item level, results are more heterogeneous: while the strongest correlation yields a p-value of 1.3e-7, three items show correlations that are not statistically significant at the 0.05 level.

**Table 2:** Results across RIASEC categories.

Category	Realistic	Artistic	Investigative	Social	Enterprising	Conventional
Correlation	0.70063	0.686256	0.503325	0.621797	0.697716	0.57577
*t*-computed	12.536	12.046	7.437	10.136	12.435	8.991
*t*_163,001_	3.351					

**Table 3:** Results across the 30 items.

Item	Self-response average	Metric average	Self-response variance	Metric variance	Correlation	*t*-computed
1	5.61	63.00	1.27	10.81	0.55	3.459
2	6.67	61.94	1.29	13.10	0.45	2.677
3	6.39	66.12	1.39	11.55	0.71	5.293
4	7.18	68.61	1.53	14.88	0.67	4.798
5	7.73	76.76	1.26	12.69	0.67	4.775
6	6.00	60.06	1.15	12.19	0.72	5.429
7	6.30	63.03	1.21	8.87	0.74	5.779
8	6.09	59.06	1.18	10.11	0.45	2.642
9	6.06	61.18	1.09	9.12	0.71	5.302
10	6.82	69.79	1.26	9.82	0.64	4.439
11	6.85	67.61	1.28	10.33	0.42	2.460
12	6.39	66.67	0.90	8.03	0.65	4.529
13	6.36	61.45	1.27	12.96	0.79	6.832
14	5.94	58.61	1.32	12.35	0.51	3.120
15	5.64	58.24	0.86	8.18	0.27	1.462
16	4.91	51.48	1.51	11.75	0.30	1.658
17	6.48	62.82	1.20	9.30	0.60	3.946
18	6.39	66.73	0.83	7.85	0.41	2.391
19	5.24	59.97	1.48	12.72	0.79	6.875
20	5.55	55.91	1.52	11.90	0.49	3.009
21	5.70	59.55	1.13	10.52	0.75	5.936
22	6.06	54.48	1.03	12.73	0.21	1.150
23	6.03	63.36	1.70	15.88	0.74	5.874
24	6.12	60.15	1.17	10.19	0.52	3.193
25	7.09	68.52	1.07	11.40	0.64	4.384
26	5.76	62.03	0.97	10.15	0.67	4.750
27	6.45	64.82	1.37	11.71	0.70	5.219
28	6.94	73.18	1.25	11.22	0.61	4.121
29	4.85	58.21	1.06	11.54	0.53	3.324
30	5.76	63.85	1.12	8.38	0.53	3.328
*t*_28,0.05_ = 2.048; *t*_28,0.001_ = 3.674						

Additional differences, with varying degrees of statistical significance, were observed between the data produced by the two methods across the 30 items in terms of average and variance scores.

## 4 Discussion

The strong correlation coefficient observed across the 990 score pairs, along with the *p*-values for individual respondents’ correlations, might suggest that using the more time-intensive EEG-based method is unnecessary. However, the relatively low rate of exact matches between RIASEC codes derived from self-reports and those obtained via EEG, combined with a closer examination of how this occupational interest inventory functions, suggests otherwise.

The findings of this research should be interpreted within the context of an exploratory pilot study involving a relatively small sample of participants (*N* = 33) (Beets et al., 2021). Consequently, although the observed relationships between EEG-derived and self-reported vocational interest profiles provide encouraging preliminary evidence, the results should be viewed as exploratory rather than conclusive. This study aimed to evaluate the feasibility of integrating EEG-based measures into vocational interest assessment and to identify trends for future investigation in larger and more diverse populations.

Even if the overall correlation does provide some comfort on the consistency in the methodology and accuracy, significant differences were recorded between different questions and different respondents, with no proven explanation so far. Therefore, attributing the differences between metric scores and self-responses among personal characteristics and question characteristics should be further investigated.

As noted before, organisations such as O*NET and the U. S. Department of Labour make substantial efforts to assess the characteristics of more than 900 occupations (Rounds et al., 2021). In this context, what may appear to be a minor discrepancy in determining an individual’s RIASEC code can actually lead to markedly different career recommendations (Gottfredson and Holland, 1996; Rounds and Tracey, 1996; Holland, 1997).

Furthermore, these organisations provide additional decision-support data, such as job outlook, median income and related factors, which, in practice, constrain the set of options a person is likely to explore (Van den Berg and Uhlendorff, 2015; National Academies of Sciences, Engineering, and Medicine, 2021; Jost and Möser, 2023; Stoica et al., 2015; Cederlöf and Roman, 2025).

Table 4 provides an example of how the slightly different profiling obtained by the two methods for one of our respondents led to quite different recommendations. It is to be taken into consideration that the search was constrained to the “zone 5” of the test, the group of occupations needing the highest level of education, according to the specific situation. If this research had enlarged the recommendations pool to all available professions in the database, the differences would have been even greater.

**Table 4:** Different occupational suggestions.

Self-response code	EEG metric code
I_SA	I_AS
Recommendations	Recommendations
Anthropologists & architects Sociologists Neuropsychologists Political scientists Marriage and family therapists	Clinical and counselling psychologists Law teachers Economics teachers Political science teachers Genetic counsellors

First of all, in Table 4. For different occupational groups, the correlation coefficient for this person’s scores was 0.79 (*p*-value = 2.087e−7, <0.001), which is considered very strong. Both the dominant category, “I,” and the three categories that form the RIASEC codes are identical, so it was decided to label this person’s code match as “strong.”

It is important to mention that only the first recommendation under the self-response code, “Anthropologists & Architects,” came with a “best fit” label from the test. In contrast, all the first five recommendations under the EEG metric were considered so.

If the direct correlation coefficient is used as a similarity index between the person’s interests and the occupation’s characteristics across all the scores and not only the first three, the result will still be impacted by the differences in assessing the person’s characteristics, which, in turn, given the high number of occupations considered, will lead to different top recommendations.

The data obtained found no significant correlation between metric—self-reported alignment and age or gender. On the other hand, large variances exist across the 30 items and the six categories. In this research, it was clear that some items show a statistically significant difference in alignment from others. Currently, researchers of this study have no data on the source of this variance. It may come from considerations of covert behaviour as well as from particular wording and translation.

The weakest alignment was recorded for item 22—“Do volunteer work at a non-profit organisation” (Social), 15—“Create special effects for movies” (Artistic) and 16—“Perform rehabilitation therapy” (Social).

Nevertheless, as an RIASEC category, the Investigative displays the lowest metric—self-reported correlation. Assessing the source of these differences was beyond the scope of this study, and for the moment, the collected data allows only for hypothesis formulation. However, properly addressing this issue may greatly improve Inventory’s accuracy in assessing respondents’ professional interests and fully benefiting from the efforts to match their profiles with various job characteristics.

Even though EEG metrics are much more effective in assessing preferences across various activities, and the Inventory’s instructions specifically provide for respondents to imagine themselves performing the corresponding activities, particular care should be taken to frame emotional content in the specific cultural context. This way, the responses EEG sensors record are elicited by the evoked activity and not by unintentional emotional content embedded in particular wording.

The findings of this study demonstrate a strong correlation between EEG-derived and self-reported RIASEC scores, while also revealing meaningful differences in the resulting vocational profiles and occupational recommendations. These results support previous studies suggesting that EEG-based measures can capture aspects of preference formation and decision-making that may not be fully accessible through self-report instruments (Khushaba et al., 2013; Lin et al., 2018; Byrne et al., 2022).

The results are also consistent with Rasheed et al. (2019), who analyzed the relationship between Holland vocational types and EEG signals, demonstrating that neurophysiological measures can be integrated into vocational assessment. Similarly, Gurres et al. (2021) identified neural correlates associated with occupational inclinations, supporting the argument that career interests have measurable neurophysiological foundations.

Unlike previous studies that focused mainly on consumer preferences or occupational tendencies, the present study directly compares EEG-based and self-reported RIASEC profiles. The observed differences suggest that EEG metrics may provide complementary insights for career guidance and could help reducing biases commonly associated with self-report measures.

These findings contribute to the growing literature in neuromarketing and consumer neuroscience by extending the use of EEG methods into educational and vocational decision-making contexts.

Other studies that support these findings can be brought into discussion. Aldayel et al. (2020) developed EEG-based preference classification models using deep learning in neuromarketing. Their findings support the idea that EEG signals can capture preferences that extend beyond self-reported responses, which aligns with the argument of this study that EEG-derived vocational profiles may provide complementary information to traditional questionnaires.

Mashrur et al. (2022) proposed an intelligent framework for predicting consumer choices from EEG signals. Similar to our study, they demonstrated that neural activity can be used to infer latent preferences and decision tendencies, reinforcing the validity of EEG-based assessment approaches.

Mizrahi et al. (2025) used EEG data to predict psychological characteristics (attachment styles). Although not focused on vocational interests, their work supports the broader premise of the present study: EEG measures can reveal underlying personal traits and preferences that may not be fully captured through self-report instruments.

### 4.1 Limitations and future research

This study aimed to investigate the usefulness of EEG-based neurometrics for identifying learners’ occupational interests, with implications for career planning and the design of educational offerings across universities and other educational service providers. Several limitations of the current research should be mentioned. However, none of them may invalidate the results and conclusions, while some may be addressed in further research.

The sample used in this research had an average age significantly higher than that of the usual students, or students-to-be. Nevertheless, no significant statistical correlation was found between the respondents’ age and the difference in profiling obtained with the two methods. It was taken into consideration from the beginning that consistently learning later in one’s career has become the norm (Guglielman, 2012; Gaydos and Kumar, 2024; Privitera et al., 2025).

Further, no attempt was made to calibrate the O*NET Mini-IP Interest Profiler to the Romanian audience. However, both the EEG scores and the self-responses were elicited by the same wording. Holland-based tools have been used in career guidance for many decades. Over the years, numerous studies have examined both the original English version and its adaptations across different countries. The model, along with many of the tools derived from it, has been thoroughly tested and is widely used in practice (Schinka et al., 1997; Petrides and McManus, 2004; Pellerone et al., 2015).

Although no systematic attempt was made to identify the source of differences between the EEG and self-reported scores, the lack of significant relationships with age or gender is encouraging (Morgan et al., 2005; Kober et al., 2016; Popov et al., 2023). Additional effort might bring insights into the split between unconscious emotional triggers and conscious covert behaviour, as some questions seem to generate larger differences than others.

One of the main limitations of this study is the relatively small sample size, which is characteristic of exploratory pilot studies. Although the sample was sufficient to identify statistically significant relationships between EEG-derived and self-reported vocational interest measures, its relatively small size restricts the broader applicability of the results. Future studies should include larger and more heterogeneous samples to validate the observed relationships, examine their robustness across different demographic groups and further assess the practical applicability of EEG-based vocational assessment.

Furthermore, because the proprietary Emotiv algorithm does not provide access to the raw EEG feature extraction and processing procedures, full independent replication of the derived “Interest (Val)” metric using different EEG metrics aiming at measuring the attractiveness of the evoked activity elicits or the alternative signal-processing frameworks is currently limited. This fact highlights the need for future validation studies based on transparent and reproducible EEG measures.

The main gap in this analysis, from our point of view, remains the comparison of respondents’ preferences for the first occupation types recommended under the two profiling methods. While previous experience demonstrated a strong declared preference for recommendations generated using EEG metrics (Pandey et al., 2020; Byrne et al., 2022), no systematic research was undertaken to document this.

## 5 Conclusion

This study achieved its intended objectives by answering the three research questions. The findings demonstrate that, despite the strong correlations between EEG-derived and self-reported vocational interest scores, the two approaches may lead to different RIASEC vocational profiles and career recommendations. This highlights the potential of EEG-based measures to improve traditional vocational assessment by capturing additional aspects of individual preferences.

While the correlation between the scores obtained by the two methods is very strong, differences do persist. Given the significant effort in assessing the characteristics of over 900 occupational types, even minor differences may lead to entirely different top recommendations for interest–occupation fit. Although each recommendation can be supplemented with a wide range of relevant information, such as average salary, career prospects, and other contextual factors, provided by organisations such as O*NET, it is difficult to provide many other relevant data for each type. As a result, the number of recommendations a user would take into consideration remains quite limited.

Given the documented superiority of EEG neurometrics over self-reported methods for assessing cognitive and emotional states, it is strongly recommended that major educational service providers incorporate these objective techniques into their assessment processes. This way, they would achieve a more accurate understanding of users’ preferences and support the development of better-tailored, more effective offerings.

The self-response recorded metric values alignment, as measured by correlation coefficients, was not significantly influenced by age and gender attributes. Variability was much greater in the correlations and in the descriptive statistics for each series across the 30 items and the six RIASEC categories. Identifying the sources of this variability and addressing them properly would substantially improve the results of applying the Inventory across both self-responses and metric-based approaches. Specifically, this study hypothesises that the wording of the questions may induce different emotional state values across social and cultural contexts, thereby interfering with responses elicited by the evoked activity, whereas the differences recorded across the RIASEC categories are arithmetic consequences.

Gottfredson and Richards (1999) bring evidence that Holland’s system of classifying academic disciplines can be understood as an ecological model (see Schoggen, 1989), because it focuses on the interaction between individuals and their environments. From this perspective, it offers a more effective way to examine how college environments influence students, as it takes into account both personal characteristics and the surrounding academic context rather than treating them separately.

This research also has important practical implications. In particular, understanding students’ personality types and intended fields of study can help practitioners design programmes that set more realistic expectations for university life. As Pike (2006) suggests, this kind of information can be used to guide learners more effectively, helping them make better-informed decisions and adjust more successfully to the demands of education providers.

Holland’s RIASEC framework can be seen as sustainable because it helps people make choices that continue to make sense over time. Instead of pushing individuals toward one fixed career, it groups interests and environments into broad categories that can apply to many different paths. This makes it especially useful in today’s fast-changing world, where jobs and skills are constantly evolving. By helping people understand what they enjoy and where they fit best, the model supports long-term satisfaction and engagement, rather than short-term decisions that may quickly become outdated (Holland, 1997; Radu et al., 2017; Savickas, 2013).

Another reason this framework can be considered sustainable is its applicability throughout a person’s life and its ability to guide ongoing learning and career changes. When people choose environments that match their interests and strengths, they are more likely to stay motivated and avoid frustration or burnout. In this way, the model supports both personal well-being and more effective education systems, helping individuals find paths that best suit them and at the same time contributing positively to society in the long term (Holland, 1997; Blustein, 2011; Savickas, 2013; Savickas and Porfeli, 2012).

Overall, this exploratory pilot study demonstrates the feasibility of combining EEG-derived measures with traditional self-report assessments to investigate vocational interests. Although the findings indicate that EEG-based metrics may provide complementary information for vocational assessment and career guidance, they should be interpreted as preliminary due to the limited sample size. Consequently, the results should not be generalized without further validation. Future research should include larger and more diverse samples, as well as longitudinal designs, to confirm these findings, test their reproducibility and further clarify the potential role of EEG-based neurometrics in vocational guidance.

## Data Availability

The raw data supporting the conclusions of this article will be made available by the authors, without undue reservation.
